# Protocol for parallel proteomic and metabolomic analysis of mouse intervertebral disc tissues

**DOI:** 10.1002/jsp2.1099

**Published:** 2020-06-10

**Authors:** Matthew A. Veras, Yong J. Lim, Miljan Kuljanin, Gilles A. Lajoie, Bradley L. Urquhart, Cheryle A. Séguin

**Affiliations:** ^1^ Department of Physiology and Pharmacology, Schulich School of Medicine & Dentistry The University of Western Ontario London Ontario Canada; ^2^ Bone and Joint Institute The University of Western Ontario London Ontario Canada; ^3^ Department of Cell Biology Harvard Medical School Boston Massachusetts USA; ^4^ Department of Biochemistry, Schulich School of Medicine & Dentistry The University of Western Ontario London Ontario Canada

**Keywords:** bioinformatics, intervertebral disc, metabolomics, proteomics

## Abstract

The comprehensiveness of data collected by “omics” modalities has demonstrated the ability to drastically transform our understanding of the molecular mechanisms of chronic, complex diseases such as musculoskeletal pathologies, how biomarkers are identified, and how therapeutic targets are developed. Standardization of protocols will enable comparisons between findings reported by multiple research groups and move the application of these technologies forward. Herein, we describe a protocol for parallel proteomic and metabolomic analysis of mouse intervertebral disc (IVD) tissues, building from the combined expertise of our collaborative team. This protocol covers dissection of murine IVD tissues, sample isolation, and data analysis for both proteomics and metabolomics applications. The protocol presented below was optimized to maximize the utility of a mouse model for “omics” applications, accounting for the challenges associated with the small starting quantity of sample due to small tissue size as well as the extracellular matrix‐rich nature of the tissue.

## 1 INTRODUCTION

The advent of “omics” technologies has transformed how biological systems are investigated, and consequently how diseases are diagnosed and treated.^1^ These approaches are particularly well suited to chronic, complex diseases such as musculoskeletal pathologies.^2^ In recent years, next‐generation sequencing‐based “omics” methodologies such as genomics and RNA‐Seq have seen a drastic increase in use due in part to standardization, including library preparation, instrumentation, and data analysis. Over time, increased use of these methodologies has resulted in a substantial reduction in cost, making them even more accessible for use by the scientific community‐at‐large.^3^

Mass spectrometry (MS)‐based “omics” methodologies such as proteomics and metabolomics are more nascent, with metabolomics being the most recent. As such, there is a lack of consensus on methodologies for sample preparation and bioinformatic analysis.^4^ Proteomics can quantitatively assess the relative abundance of thousands of proteins in a tissue or circulating in plasma with the sensitivity and dynamic range to allow for high‐throughput biological insights and biomarker discovery.^5^ For instance, proteomics has been used to identify biomarkers of mortality in older men,^6^ characterize the effects of sustained weight‐loss,^7^ determine the composition of cartilage,^8^ and how cartilage responds to injury and inflammation.^9^ Metabolomics allows for the comprehensive analysis of all small molecule metabolites in a diseased tissue or in circulation to develop a functional readout of the pathological state of an organism.^1^ This technology has been used to develop biomarkers and therapeutic targets for numerous disorders including diabetic nephropathy, renal failure, cardiovascular disease, and prostate cancer.^2^ Since MS‐based proteomic and metabolomic techniques can be used for many sample types, it can be difficult to standardize instrument parameters, therefore optimization must be done on a sample‐by‐sample basis. Moreover, metabolomic techniques have been difficult to standardize due to differences in physicochemical properties of metabolites. The differences in metabolite properties may necessitate the use of several techniques (eg, LC‐MS, GC‐MS, NMR) to be fully comprehensive. However, the comprehensive data collected by these “omics” modalities has the potential to drastically transform our understanding of the molecular mechanisms of disease, how biomarkers are identified, and how therapeutic targets are developed. Therefore, it is essential that standardized protocols be developed within specific fields of research to enable comparisons between findings reported by multiple research groups and move the application of these technologies forward.

One such field of research is intervertebral disc (IVD) biology, which lacks standardization of disease models and model organisms. Numerous models of common spine pathologies such as IVD degeneration have been reported, induced by aging,^10^ mechanical loading (ie, compression^11^ or tail‐loop^12^), surgical injury,^13^, ^14^, ^15^ or genetic manipulation.^16^, ^17^, ^18^, ^19^ Many of these models have been validated in multiple model organisms including cow, pig, sheep, goat, dog, rat, and mouse, for which each has advantages and disadvantages.^20^ The lack of standardization in IVD biology can also be exemplified by recent efforts to determine a standardized set of cell type‐specific phenotypic markers. Cell type‐specific markers have been proposed either by focusing on specific candidate genes or classes in human primary cells^21^, ^22^, ^23^, ^24^, ^25^ and animal models,^25^, ^26^, ^27^, ^28^, ^29^ or by using unbiased whole transcriptome approaches.^30^, ^31^, ^32^, ^33^, ^34^, ^35^, ^36^, ^37^, ^38^, ^39^ Although these studies have identified a common subset of markers that have helped to define IVD cell phenotypes, their findings also highlight differences across species.^34^, ^39^, ^40^, ^41^ This heterogeneity in model systems and methodologies is not ideal for exploring disease mechanisms at the molecular level that can then be translated to the clinic.

For experimental strategies exploring the molecular mechanisms of disease, or seeking to identify disease biomarkers or therapeutic targets, a key model organism is the mouse. This is due to the relatively short gestation period and lifespan, ease of genetic manipulation, and robustness of bioinformatic databases compared to those of other organisms. Work by our group and others have established the strength of mouse models to study IVD biology and common spine disorders. We have explored the effect of mechanical loading on the IVD in mice^42^, ^43^, ^44^ and used transgenic mice to study IVD development,^45^ disc degeneration,^16^, ^46^ and diffuse idiopathic skeletal hyperostosis (DISH).^47^, ^48^, ^49^, ^50^ Important insights have likewise been provided by others using mouse models to study disc development,^51^, ^52^, ^53^ inflammation,^54^ IVD degeneration,^10^, ^13^, ^55^, ^56^, ^57^, ^58^ calcification,^59^, ^60^ and scoliosis.^61^, ^62^, ^63^ The multitude of available mouse models of spine pathologies allows for global molecular comparisons to uncover novel biological insights. The use of unbiased “omics” approaches increases the likelihood of uncovering novel pathways implicated in spine pathologies, and therefore candidate targets for therapeutic interventions and novel biomarkers.

Transcriptomics technologies have been applied extensively to study the IVD, identifying numerous genes associated with IVD degeneration in model organisms^37^, ^39^, ^46^, ^64^, ^65^ as well as humans.^66^, ^67^, ^68^, ^69^ In comparison, the use of proteomics has been limited, with a few studies of IVD degeneration in humans,^70^, ^71^, ^72^ although access to tissues at various stages of diseases is limited. Proteomics has also been used in mice for global characterization of the healthy IVD,^73^ to examine the response to mechanical loading,^42^ to characterize different mouse strains,^55^ and to investigate ectopic calcification in the IVD.^48^ To date, there have been no metabolomic studies of murine IVD tissues, and the analysis of human IVD tissues is limited to a single unbiased metabolite screen using high‐resolution magic angle spinning (HR‐MAS) nuclear magnetic resonance (NMR),^74^ which is much less sensitive than MS based metabolomics.^75^ Despite the limitations to the starting quantity of sample associated with the small size of the mouse, it is possible to gain novel insights into mechanisms, biomarkers and therapeutic targets of IVD pathologies using optimized protocols for proteomic and metabolomic analyses.

**FIGURE 1:**
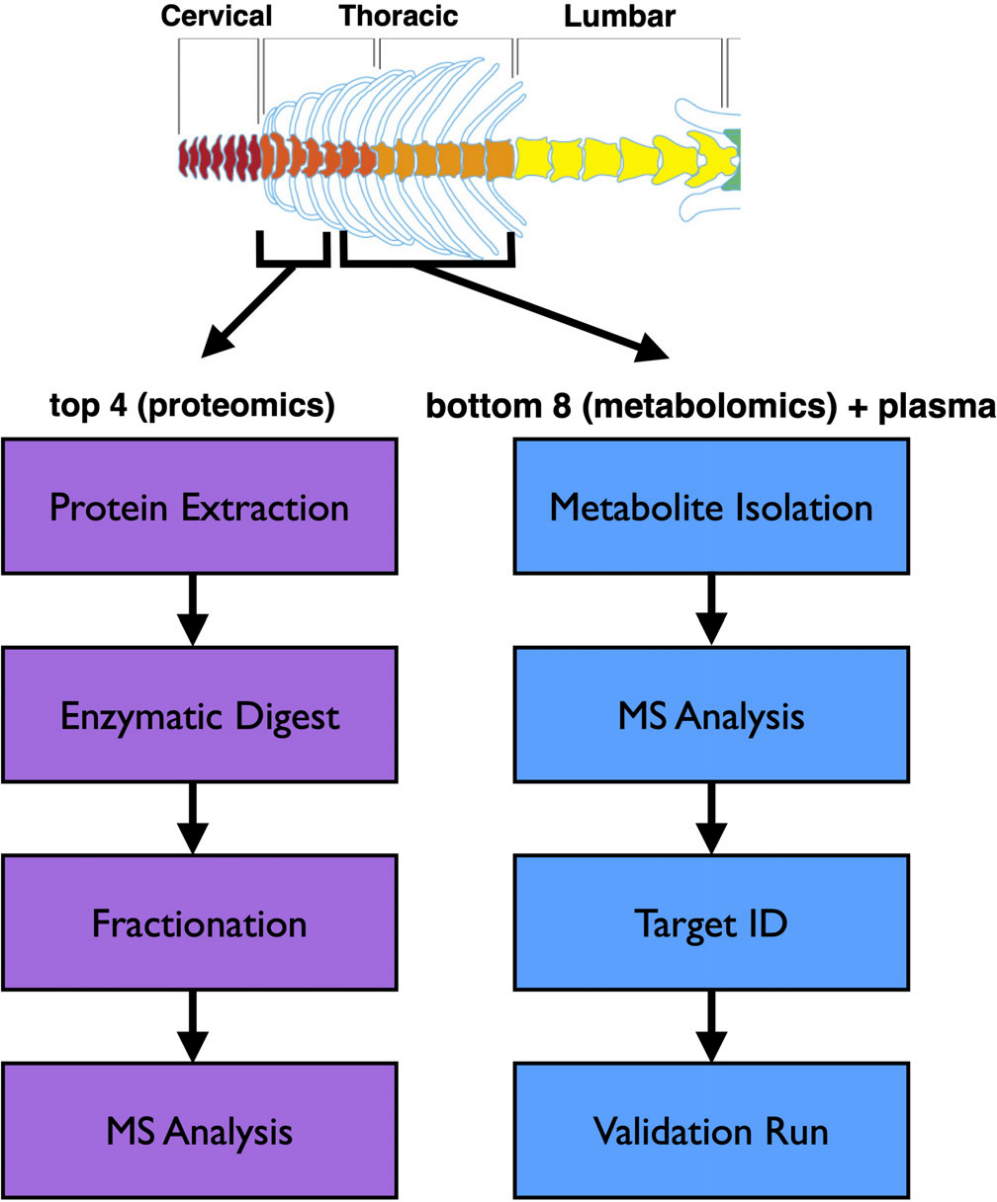
Schematic overview of protocol for simultaneous assessment of proteomic and metabolomic changes

Herein, we describe a protocol for parallel proteomic and metabolomic analysis of mouse IVD tissues, building from the combined expertise of our collaborative team (Figure 1). Our group has lead technical development in proteomics^76^, ^77^, ^78^, ^79^, ^80^ and applied these methodologies to develop biomarker panels to improve classifications of ovarian carcinomas,^81^ evaluate the potential of multipotent stromal cells for pancreas regeneration,^82^ and developed optimized protocols to characterize extracellular matrices,^83^, ^84^, ^85^ or increase the detection of low‐abundant proteins in extracellular matrix‐rich samples.^86^ These studies led to the development of the current protocol for label‐free quantitative proteomics of murine IVD tissue.^48^ Our group has also used metabolomics to develop biomarkers of chronic kidney disease,^87^ muscle response to exercise in diabetes,^88^ kidney function,^89^ and characterize the association of the microbiome to atherosclerosis.^90^ The metabolomics methods used previously to assess kidney and muscle tissues required minimal adaptation for use with murine IVD tissue.

The protocol presented below was optimized to maximize the utility of a mouse model for “omics” applications, accounting for the challenges of minimal starting quantity of sample due to small tissue size as well as the extracellular matrix‐rich nature of the tissue. This protocol could be used to standardize tissue isolation, sample preparation, fractionation, and run parameters to allow comparative analysis between datasets generated from different research groups using mouse models to study IVD biology. The protocol was developed to investigate proteomic and metabolomic changes in annulus fibrosus tissue from the thoracic spine of a transgenic mouse model of diffuse idiopathic skeletal hyperostosis (DISH). For these analyses, the methods are expected to be robust and reproducible. While the methodologies are sensitive, a primary limitation is obtaining enough tissue from each sample (discs will have to be pooled) and ensuring complete homogenization of small fibrous tissues. Incomplete homogenization will result in a lower number of features being detected and ultimately less peptides and metabolites being identified. Importantly, for metabolomics, prior to experimentation, ensure the detector, lockspray and calibration setups have been performed and the sample cone has been cleaned by sonication in formic acid. In addition, our method will detect molecules with *m/z* between 50 and 1200. Features greater than 1200 *m/z* will not be captured.

For our specific experimental question, the protocol was designed to isolate protein and metabolites from the annulus fibrosus of IVDs within a specific anatomical region from each mouse; sample isolation should therefore be more straightforward for experiments that allow for pooling of IVDs from multiple anatomical regions or those focused only on one type of analysis. This protocol is not limited to a particular genetic background of mouse as IVD size is generally consistent across strains. Furthermore, this protocol is not limited to thoracic IVDs as lumbar and ‐caudal IVDs would be even larger, and thus easier to isolate samples, though cervical IVDs may be challenging. Theoretically, this protocol could be used for NP tissue, however, there are typically fewer NP cells compared to AF cells in an IVD, so tissue yield by weight (or total protein for proteomics) would need to be equivalent to AF, requiring the use of more IVDs. However, using a whole IVD should not present any challenges compared to use of AF alone. Furthermore, these methodologies should be applicable to other small rodent models such as the rat, as tissue composition is very similar, and tissues are even larger.

## 2 ANTICIPATED RESULTS

**FIGURE 2:**
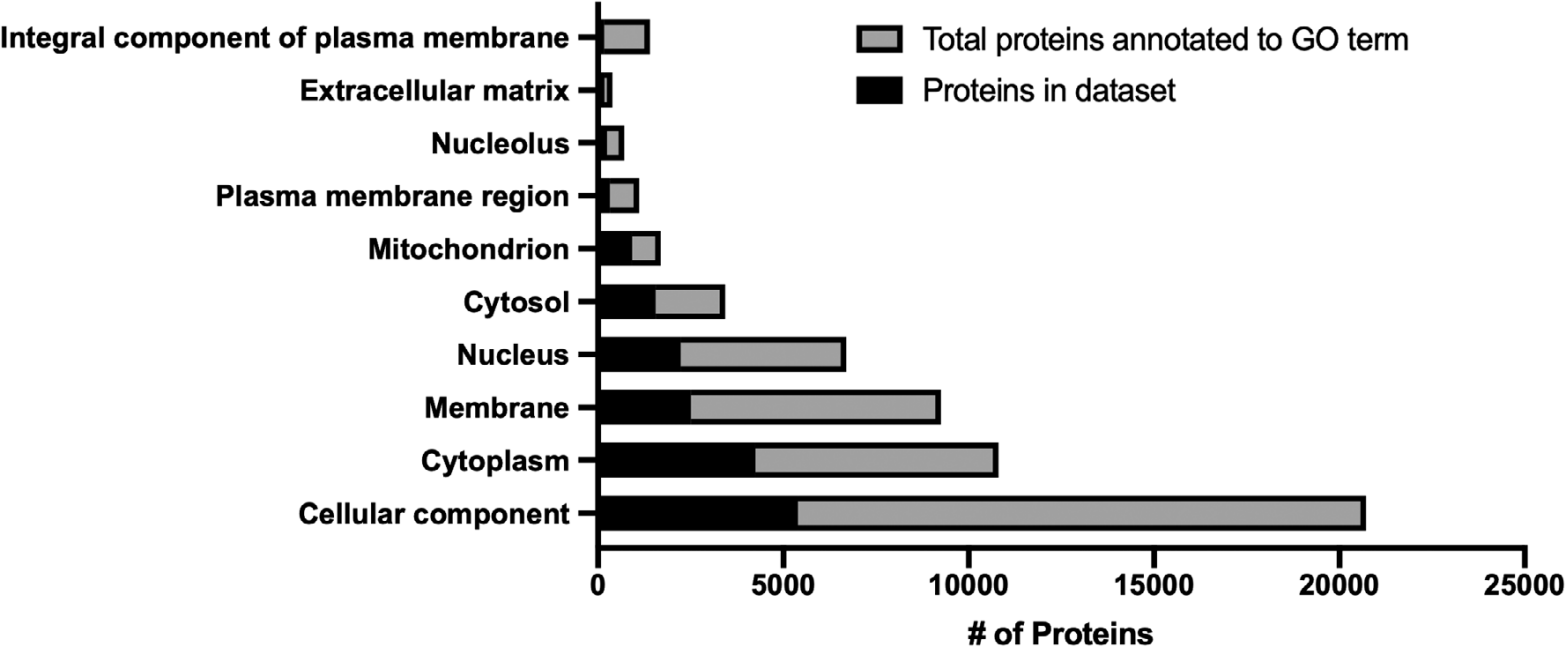
Gene ontology (GO) cellular component enrichment for proteomics. Proteins quantified in all experimental groups (black bars) are overlaid on the total proteins annotated to a given GO term (gray bars) for various cellular components. n = 3 mice/genotype and age combination (2 mo WT, 2 mo KO, 6 mo WT, 6 mo KO)

*Proteomics*: Based on our experiments using 2‐ or 6‐month‐old C57/Bl6 mice, AF tissues isolated from 4 thoracic IVDs should yield at least 1.5 mg of tissue (wet weight). This should lead to a total protein yield between 40 and 80 μg per sample, of which 25 μg is needed per run. With fractionation, this proteomics protocol consistently quantified >5000 unique proteins per sample (98% of identified proteins were quantified), a greater than 2‐fold increase in detection compared to our previous method that did not use fractionation and used a different MS instrument^73^ (Table 1). Importantly, proteins are detected from all cell compartments (cytoplasm, nucleus, mitochondrion, plasma membrane, extracellular matrix) (Figure 2), suggesting this protocol reduces potential bias of high‐abundance extracellular matrix proteins in IVD tissue that could obscure low‐abundance intracellular proteins.

**TABLE 1:** Number of unique proteins identified and quantified by proteomics

Sample	# unique proteins identified	# unique proteins quantified	% quantified
2 mo WT 1	5527	5404	97.8
2 mo WT 2	5418	5340	98.6
2 mo WT 3	5501	5408	98.3
2 mo KO 1	5390	5306	98.4
2 mo KO 2	5164	5095	98.7
2 mo KO 3	5294	5224	98.7
6 mo WT 1	4725	4669	98.8
6 mo WT 2	5314	5216	98.2
6 mo WT 3	4334	4216	97.3
6 mo KO 1	4825	4709	97.6
6 mo KO 2	5077	4970	97.9
6 mo KO 3	5188	5060	97.5
Average	5146	5051	98.2

Method	# unique proteins identified
McCann et al., 2016	1940
Veras et al., 2020	5146
Fold‐increase in coverage	2.65‐fold

*Metabolomics*: Due to the low concentration of metabolites in the IVD compared to plasma, the 300 μL of disc metabolite preparation (generated from eight thoracic AFs; ~ 3 mg of tissue) will allow for 2 to 3 metabolomics runs, if samples are pooled for a validation run with analytical standards. The disc metabolite preparation will not yield high‐quality results after 6 months of being stored at −80°C (loss of signal intensity due to low starting quantity), but plasma samples will last over 1 year (retaining the same signal intensity). Based on this protocol, detection of >300 features should be expected in both plasma and IVD samples (Table 2). However, it is important to carefully consider sample size for metabolomics studies, as abundance of metabolites is often highly variable even within sample groups. We suggest a minimum of 10 biological replicates stratified by sex to reduce sex‐related differences in metabolite abundance. Metabolomics validation is also critical, whereby one should aim for level 1 validation by analytical standard of at least five metabolites‐of‐interest based on criteria developed in the field.^91^

**TABLE 2:** Number of features detected by metabolomics and number meeting threshold values (fold‐change ≥2 or ≤−2, *P* < .05)

Sample	# unique metabolites identified
Plasma	314
IVD	714

Comparison	# unique metabolites meeting VIP and pcorr thresholds
6 mo WT vs 6 mo KO Plasma	29
6 mo WT vs 6 mo KO IVD	29
2 mo WT vs 2 mo KO Plasma	24
2 mo WT vs 2 mo KO IVD	42
2 mo KO vs 6 mo KO Plasma	28
2 mo KO vs 6 mo KO IVD	41
2 mo WT vs 6 mo WT Plasma	22
2 mo WT vs 6 mo WT IVD	88

Overall, the use of this protocol should be expected to provide additional biological insights into molecular mechanisms of spine pathologies, biomarker development, and therapeutic targets beyond the use of genomics or transcriptomics alone. Furthermore, the acquisition of data by multiple “omics” modalities from the same animal allows for integration of multiomics datasets in the future when more powerful bioinformatic tools are developed.

## 3 MATERIALS

### 3.1 Reagents

#### 3.1.1 Proteomics

**Ammonium acetate** (Sigma‐Aldrich, cat. no. 73594)

**Ammonium bicarbonate solution**:

Prepare 50 mM ammonium bicarbonate solution by dissolving ammonium bicarbonate (Sigma‐Aldrich, cat. no. 09830) in HPLC‐grade water. This solution can be made ahead of time and stored up to 2 months at room temperature (RT).

**8 M urea protein extraction buffer**:

Prepare protein extraction buffer by mixing 50 mM ammonium bicarbonate, 2% sodium dodecyl sulfate (SDS; Sigma‐Aldrich, cat. no. L6026) and 8 M urea (Sigma‐Aldrich, cat. no. 05378) at pH 8.0. The buffer should be freshly prepared.

**DTT reducing solution**:

Prepare 100 mM dithiothreitol (DTT) solution by dissolving DTT (Sigma‐Aldrich, cat. no. D9779) in HPLC‐grade water. This solution should be made fresh to prevent self‐quenching of DTT after solubilization.

**IAA alkylating solution**:

Prepare 100 mM iodoacetamide (IAA) solution by dissolving IAA (Sigma‐Aldrich, cat. no. I1149) in HPLC‐grade water. This solution should be made fresh and protected from light.

**LC solvent A**:

LC solvent A is 99% (vol/vol) MS‐grade water, 1% (vol/vol) MS‐grade acetonitrile (EMD Millipore, cat. no. AX1056‐1) and 0.1% (vol/vol) MS‐grade formic acid (FA; EMD Millipore, cat. no. 1002641000). This solution can be stored at RT for up to 1 year.

**LC solvent B**:

LC solvent B is 99% (vol/vol) MS‐grade acetonitrile, 1% (vol/vol) MS‐grade water and 0.1% (vol/vol) MS‐grade formic acid (FA). This solution can be stored at RT for up to 1 year.

**Lys‐C Aliquot** (FUJIFILM Wako Pure Chemical Corporation, cat. no. 121‐05063, 20 μg quantity):

Prepare Lys‐C aliquot at a concentration of 0.04 μg/μL in 50 mM ammonium bicarbonate (ABC) by dissolving 20 μg of enzyme in 500 μL ABC. This can be stored at −20°C for up to 1 year.

**Trypsin/Lys‐C Aliquot** (Promega, cat. no. V5071, 20 μg quantity):

Prepare Trypsin/Lys‐C aliquot at a concentration of 0.04 μg/μL in 50 mM ammonium bicarbonate (ABC) by dissolving 20 μg of enzyme in 500 μL ABC. This can be stored at −20°C for up to 1 year.

#### 3.1.2 Metabolomics

**Atenolol‐d7** (Toronto Research Chemicals, cat. no. A790077, 10 mg quantity):

Prepare a stock solution at a concentration of 1 mg/mL in 50:50 methanol:water (vol/vol, LC‐MS grade). This can be stored at −20°C for up to 1 year.

**Chlorpropamide** (Sigma Aldrich, cat. no. C1290, 25 g quantity):

Prepare a stock solution at a concentration of 1.38 mg/mL in 50:50 methanol:water (vol/vol, LC‐MS grade). This can be stored at −20°C for up to 1 year.

**DL‐2‐aminoheptanedioic acid** (Bachem, cat. no. 4014992, 5 g quantity):

Prepare a stock solution at a concentration of 8.75 mg/mL in LC‐MS grade water. This can be stored at −20°C for up to 1 year.

**Flurazepam** (Cerilliant, cat. no. F003, 1 mL at 1.0 mg/mL)

**Formic acid** (EMD Millipore, cat. no. 1002641000, 1 L bottle)

**LC‐MS grade acetonitrile** (EMD Millipore, cat. no. AX1056‐1)

**Milli‐Q water** (or other LC‐MS grade water) RT

## 4 EQUIPMENT

#### 4.1.1. Proteomics

**UPLC system**: Waters nanoACQUITY UPLC M‐Class.

**Mass spectrometer**: ThermoScientific Q Exactive Plus.

**Software**: MaxQuant 1.5.0.30 and Perseus 1.5.0.8 software packages used for data analysis.

**Columns**:

Trapping column: Waters ACQUITY UPLC M‐Class Symmetry C18 Trap Column (100 Å pore size, 1.7 μm particle size, 25 cm × 75 μm; SKU: 186007496).

Analytical column: Waters ACQUITY UPLC Peptide BEH C18 column (130 Å pore size, 1.7 μm particle size, 25 cm × 75 μm; SKU: 186003556), set column temperature to be maintained at 35°C.

#### 4.1.2. Metabolomics

**UPLC System**: Waters ACQUITY UPLC I‐Class.

**Mass spectrometer**: Waters Xevo G2‐S QTof.

**Software**: Waters MassLynx 4.1.1 software package used for data acquisition and control of UPLC and MS parameters.

Umetrics EZinfo 2.0 software package (must be purchased) for statistical analysis of metabolomics data.

**Column**: Waters ACQUITY UPLC HSS T3 (100 Å pore size, 1.8 μm particle size, 100 mm × 2.1 mm; SKU: 186003539) reverse‐phase C18 column, set column temperature to be maintained at 45°C.

## 5 EQUIPMENT SET‐UP/CONDITIONS

### 5.1 Proteomics

#### 5.1.1 Liquid chromatography

Use the following LC gradient at a flow rate of 0.3 mL/min for whole proteome analysis (Data acquisition and analysis section: Proteomics, step 2):

*Note:* Gradients should be optimized for each specific column type, LC, and solvent setups.

**Table:** 

Time interval (min)	LC solvent A (%)	LC solvent B (%)
0	95	5
74	67.5	32.5
80	40	60
81	5	95
90	5	95

#### 5.1.2 MS analysis

Settings for whole proteome analysis on a Q Exactive Plus MS instrument are described in the following table.

*Note:* Instrument parameters may need to be adjusted slightly due to variability in performance between MS instruments.

**Table:** 

Method parameter	Value
Polarity	Positive
Mass range (*m/z*)	400‐1500
Micro scans	1
Resolution	70 000 @ 200 *m/z*
AGC target	3E6
Maximum injection time (ms)	250
dd‐MS^2^	
Micro scans	1
Resolution	17 500 @ 200 *m/z*
AGC targets	2E5
Maximum ion time (ms)	64
Loop count	1
Isolation window (*m/z*)	2
Isolation offset (*m/z*)	0
Fixed first mass	100
Normalized collision energy	25
Data dependent acquisition	Top12
Threshold (counts)	2E3
Minimum AGC target	3.1E4
Peptide match	Preferred
Exclude isotopes	Enabled
Fragmentation type	HCD
Charge state rejection	Unassigned, +1, 7, >8
Lock mass (445.120025 *m/z*)	Best
Dynamic exclusion (s)	30

### 5.2 Metabolomics

For the Waters ACQUITY system, set injection volume of 2 μL for plasma metabolomics, 5 μL for disc tissue metabolomics. Randomize the sample injection order for both plasma and disc runs.

Gradient conditions are as follows.

#### 5.2.1 Gradient conditions

**Table:** 

Time (min)	%*B*	Flow rate (mL/min)
0.0	1	0.45
2.0	60	0.45
6.0	85	0.45
8.0	99	0.45
10.0	1	0.45
11.0	1	0.45

#### 5.2.2 MS parameters and data acquisition

*Note:* Instrument parameters may need to be adjusted slightly due to variability in performance between MS instruments.

Measure metabolites in both positive and negative electrospray ionization (ESI) modes with the following MS instrument conditions:

Capillary voltage: 2.00 kV.
Cone voltage: 40 V.
Source temperature: 150°C.
Desolvation gas flow: 1000 L/h.
Desolvation gas temperature: 500°C.
Cone gas flow: 50 L/h.

Set MS acquisition settings to acquire data in centroid, using the MS^E^ method in resolution mode. The MS^E^ method allows for the simultaneous generation of precursor (function 1 of MS^E^ method) and fragment ions (function 2 of MS^E^ method) in a single run. The acquisition period is 11 minutes, with 0.05 second scan time and a mass range of 50 to 1200 Da. Set collision energy as 0 V for function 1 and ramp the collision energy from 15 to 50 V for function 2. Use leucine‐enkephalin (500 ng/mL) as the lockspray solution to ensure mass accuracy. Infuse the lockspray solution at a flow rate of 10 μL/min. Set the lockmass to be acquired at intervals of 10 seconds and averaged over three scans.

## 6 SAMPLE COLLECTION

All aspects of this study were conducted in accordance with the policies and guidelines set forth by the Canadian Council on Animal Care and were approved by the Animal Use Subcommittee of the University of Western Ontario (protocol 2017‐154).

### 6.1 Blood plasma collection (timing: ~ 5 minutes per mouse)

1. Precool microcentrifuge to 4°C.
2. Weigh mice and prepare sodium pentobarbital at a concentration of 270 mg/mL to be used at a 540 mg/kg dosage.
3. Prepare 25‐gauge blood drawing needles with heparin (Sandoz, cat. no. 10750) coating.
4. Administer sodium pentobarbital (Bimeda‐MTC, cat. no. 8EUT002) by intraperitoneal injection.
5. Once the breathing of the mouse is slowed, perform cardiac puncture and withdraw blood, aiming to collect 500 μL to 1 mL for an adult mouse.
6. Immediately transfer blood to 1.5 mL microcentrifuge tube and centrifuge at 4000 RPM for 10 minutes at 4°C to separate plasma from other phases.
7. Immediately transfer plasma supernatant (taking care to avoid the interphase) to a new 1.5 mL microcentrifuge tube. Immediately freeze at −80°C.

### 6.2 IVD microdissection (timing: ~30‐45 minutes per mouse)

**FIGURE 3:**
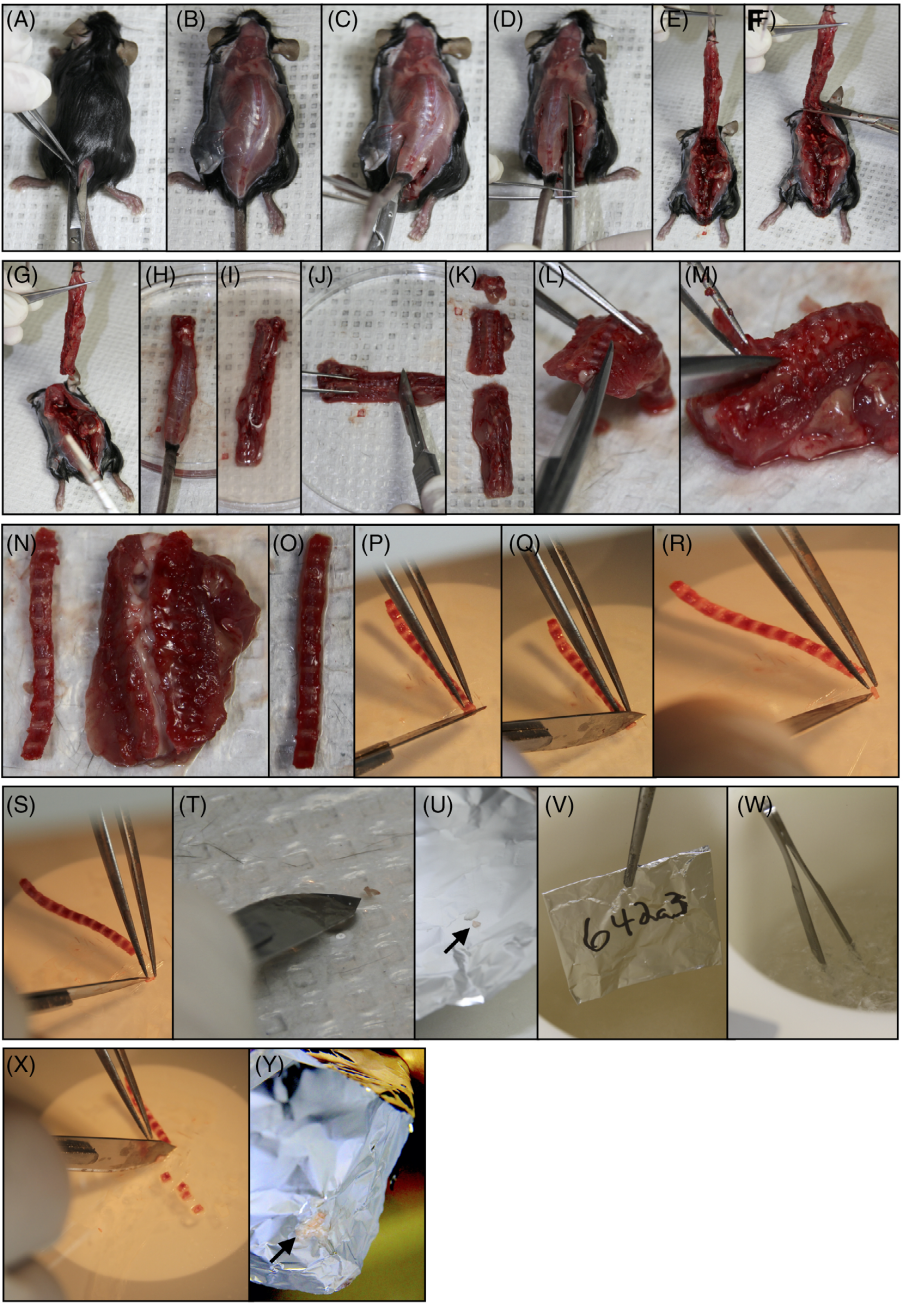
Intervertebral disc (IVD) microdissection. A‐G, Removal of spinal column. H‐K, Isolation of thoracic spine. L‐O, Dissection of anterior thoracic spine. P‐U, IVD microdissection and AF isolation. V and W, Snap freezing in liquid nitrogen. X and Y, Repetition of previous steps for entire thoracic spine

1. Fill dewar with liquid nitrogen and fold aluminum foil to create pouches for each mouse sample (one for metabolomics, one for proteomics). Label aluminum foil directly with permanent marker (adhesive tape will fall off in liquid nitrogen).
2. Following cardiac puncture, rotate mouse to expose the dorsal side and douse fur with 70% ethanol in H_2_O (vol/vol). Make an incision with scissors (length: 11.5 cm; cutting edge: 25 mm) just above the tail, taking care to cut only through the skin, and continue incision up to the skull (Figure 3A,B).
3. Gently peel back skin to expose the spinal column and then make incisions on either side of the lumbar spine with scissors (through musculature and surrounding tissues) and cut along each side of the spine to the base of the skull (Figure 3C,D).
4. Holding at the base of the tail, lift spine from the body of the mouse (Figure 3E) to make a transverse cut through the spinal column at the base of the skull (Figure 3F) to remove the intact spinal column and rinse in PBS (Figure 3G,H).
5. Make transverse cuts within the sacral region (Figure 3I) below the bottom rib (Figure 3J) and above the top rib to isolate the cervical, thoracic, and lumbar spines (Figure 3K).
*Note:* The remaining protocol focuses on the annulus fibrosus of the thoracic region, but techniques should be applicable to NP or intact IVD from any anatomical region. Furthermore, the caudal region can be used in the same manner as long as the skin and ligaments are removed. However, at least 3 mg of disc tissue is needed to detect metabolites. Proteomics would require a minimum of 1.5 mg of disc tissue (yielding 40‐80 μg of protein).
6. Isolate the anterior aspect of the thoracic spine (vertebrae + IVDs) by inserting scissors into the vertebral foramen (Figure 3L) and cutting along the length of the spinal column directly adjacent to IVD bilaterally to remove all musculature, connective tissue and spinous processes (Figure 3M,N).
7. Use scalpel to scrape off as much soft tissue as possible surrounding the vertebrae and IVDs (Figure 3O).
8. Use stereoscope to microdissect each individual IVD by using transverse cuts where the vertebral body meets the IVD on the inferior (Figure 3P) and superior (Figure 3Q) side to isolate the intact IVD.
9. Once an IVD is isolated, use a scalpel to scrape away the hard, cartilaginous endplate from both surfaces (Figure 3R) and then lacerate the AF on one side (Figure 3S) and place IVD briefly into PBS to allow the NP to leak out (Figure 3T; as previously reported^46^). Scrape along inner AF to remove any remaining NP tissue.
10. Quickly transfer the AF to aluminum foil pouch (Figure 3U,V) and immediately snap freeze in liquid nitrogen (Figure 3W). Repeat to collect a total of four thoracic AFs adding each to a single pouch for proteomics. Repeat to collect an additional eight thoracic AFs into a single pouch for metabolomics (Figure 3X,Y).
**CRITICAL STEP**: Leave AF tissue in PBS for as little time as possible (~1‐2 seconds), only to allow NP to leak out, as PBS can contaminate mass spectrometer at high concentrations and metabolites can diffuse out from the tissue into PBS.
11. Once all tissues are snap frozen, remove from liquid nitrogen and weigh to ensure a minimum of 1.8 mg of tissue for metabolomics and 1 mg for proteomics (corresponding to ~40‐80 μg of protein).
12. Immediately transfer tissue to 1.5 mL low‐retention microcentrifuge tubes and store at −80°C until sample preparation (up to 6 months).

## 7 PROTEOMICS

### 7.1 Protein extraction (timing: ~2 hours)

**FIGURE 4:**
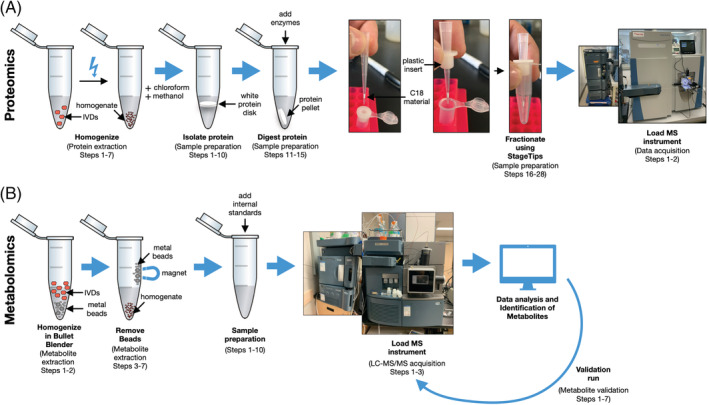
Schematic overview of workflow for proteomics and metabolomics. A, Proteomic workflow including homogenization (protein extraction steps 1–7), protein isolation (sample preparation steps 1‐10), protein digestion (sample preparation steps 11‐15), fractionation (sample preparation steps 16‐28), and the mass spectrometry instrument. B, Metabolomic workflow including homogenization (metabolite extraction steps 1 and 2), metal bead removal (metabolite extraction steps 3‐7), addition of internal standards (sample preparation steps 1‐10), validation run (metabolite validation steps 1‐7), and the mass spectrometry instrument

Overview of proteomics is provided in Figure 4A.

1. Prepare protein extraction buffer (8 M urea, 50 mM ammonium bicarbonate, 10 mM dithiothreitol, and 2% SDS). Buffer should be prepared fresh.
2. Mince each IVD into approximately 8 pieces prior to homogenization.
3. Add 100 μL of extraction buffer to each sample and sonicate to homogenize at an intensity of 1 (Sonic Dismembrator Model 100, Fisher Scientific) using 25 pulses spaced 1 second apart.
**CAUTION**: Always wear protective ear equipment while sonicating. Also, be careful to sonicate in bursts to avoid melting the sample tube. Excessive heating of the samples in 8 M urea extraction buffer will results in peptide carbamylation. Alternatively, sonication can be done in a cold room or on ice to minimize heating.
4. Clarify lysates by centrifugation at 16 000 RCF for 5 minutes at RT and transfer supernatant to new 1.5 mL low‐retention microcentrifuge tubes (Fisher Scientific, cat. no. 02‐681‐320).
**CRITICAL STEP**: Always use low‐retention tubes to ensure complete recovery and minimize loss of peptide or proteins during MS sample preparation.
5. Measure protein concentration (in triplicate) using the Pierce 660 nm protein assay (ThermoFisher Scientific, cat. no. 22662) with a CLARIOstar Plus plate reader (BMG Labtech).
**CRITICAL STEP**: Pierce 660 nm assay must be used since DTT will interfere with quantification of protein concentrations when using the BCA assay.
**PAUSE POINT**: Samples may be stored at −80°C indefinitely for future use.
6. Reduce protein extracts using 10 mM DTT for 30 minutes at RT with thorough vortexing.
7. Alkylate protein extracts with 20 mM iodoacetamide for 30 minutes at RT in the dark with thorough vortexing.
**PAUSE POINT**: Samples can be stored at −80°C for up to 1 year.

### 7.2 Sample preparation (timing: 24‐48 hours)

1. Transfer 25 μg of protein per sample into new 1.5 mL low‐retention microcentrifuge tubes for precipitation according to the Wessel and Flügge protocol.^92^
**CRITICAL STEP**: It is imperative to keep all reagents cold for precipitation. Refrigerate methanol and chloroform until use.
2. Dilute samples with HPLC‐grade H_2_O to total volume of 150 μL.
3. Add 150 μL cold chloroform (BioShop, cat. no. CCL4027) and 650 μL methanol (Fisher Scientific, cat. no. A412) and vortex vigorously.
4. Fill remaining volume in 1.5 mL low‐retention microcentrifuge tubes with H_2_O and vortex vigorously.
**CRITICAL STEP**: Protein precipitate should be visible as an opaque or white solid. If proteins are not visible, the ratios of solvents need to be adjusted by adding more HPLC‐grade water.
5. Centrifuge at 14 000 RCF for 5 minutes at RT.
6. Preheat ThermoMixer to 37°C.
7. Remove the top aqueous layer from samples and discard.
**CRITICAL STEP**: A white protein “disk” should be visible near the interface between the chloroform and methanol‐water layer.
8. Wash the protein pellet by filling sample tubes with methanol and vortexing vigorously.
9. Centrifuge at 14 000 RCF for 5 minutes at RT.
10. Remove the top aqueous layer from samples and discard (white protein “disk” should still be visible). Leave sample tubes open on benchtop for 5 minutes to evaporate all excess methanol.
**CRITICAL STEP**: The protein pellet can be weakly attached to the walls of the centrifuge tube. Take care in removing the final chloroform‐methanol solution. If a protein pellet is not firmly formed against the walls of the centrifuge tube, centrifuge again at 14 000 RCF for 5 minutes.
11. Add 100 μL 50 mM ABC to each sample containing 6.25 μL Lys‐C (1:100 enzyme to protein ratio vol/vol) and 12.5 μL Trypsin/Lys‐C (1:50 enzyme to protein ratio vol/vol).
**CRITICAL STEP**: Do not touch protein pellet at this step as it will stick to the pipette tip and significant sample loss will occur. Ensure the pellet is removed from the side of the tube by a short vortex to ensure maximum surface area exposure to enzymes.
12. Place samples in ThermoMixer at 37°C overnight (approximately 18 hours) at 700 RPM.
13. Add an additional aliquot of 6.25 μL Trypsin/Lys‐C (1:100 enzyme to protein ratio vol/vol) to each sample and continue mixing in ThermoMixer at 37°C at 700 RPM for an additional 4 hours.
14. Acidify samples with 10% formic acid (pH 3‐4) and centrifuge at 14 000 RCF for 2 minutes. Remove supernatant and transfer to new 1.5 mL low‐retention microcentrifuge tubes.
**CRITICAL STEP**: Centrifugation must be done following acidification to remove insoluble material that will interfere with injection into the mass spectrometer.
15. Quantify peptide concentrations with the Pierce BCA assay (ThermoFisher Scientific, cat. no. 23250).
**PAUSE POINT**: Samples can be stored at −80°C for up to 1 year.
16. Transfer approximately 20 μg of tryptic peptides to a new 1.5 mL low‐retention microcentrifuge tube for basic reversed‐phase (RP) fractionation (bRP).
**CRITICAL STEP**: Due to the low starting quantity of sample material, to maximize the number of quantifiable peptides, basic reversed‐phase fractionation (bRP) must be performed. This helps reduce bias of increased detection of matrix proteins in an extracellular matrix‐rich tissue and allows detection of proteins from all cell compartments.
17. Prepare StageTips by punching 12 discs of C18 using an 18‐gauge needle and packing a 200 μL StageTip.
18. Place tips into centrifuge and add 50 μL ACN to each tip and centrifuge at 10 000 RCF at RT for 2 minutes. Discard flow through.
**CRITICAL STEP**: Flow through may vary, so be sure not to dry out C18 completely. A dry C18 will not retain peptides properly. Adjust speeds and time according to centrifuge.
19. Prepare elution buffers containing 25 mM ammonium acetate (pH 10) and increasing the % of ACN for each of 7 fractions as follows: (a) 5%; (b) 7.5%; (c) 10%; (d) 12.5%; (e) 15%; (f) 17.5%; (g) 50%. These solutions can be stored for up to 1 year at RT.
20. Add ammonium acetate in 50% ACN to tips and centrifuge at 1000 RCF until all liquid flows through (approximately 2‐3 minutes). Discard flow through.
21. Add 100% ammonium acetate to tips to equilibrate and centrifuge at 1000 RCF until all liquid flows through (approximately 2‐3 minutes). Discard flow through.
22. Turn on condenser SpeedVac (ThermoFisher Scientific) to cool (minimum 30 minutes prior to use). Preheat centrifuge attached to condenser to 60°C to be ready when fractionation is complete.
23. Resuspend peptide sample in 100 μL of ammonium acetate to ensure the pH is ~10. Load samples onto the stage tips for centrifugation and to prepare for elution.
**CRITICAL STEP**: pH at least 1 sample at this step to ensure it is ~pH 10. Our samples were around pH 2 to begin, and pH 10 afterward. Determine pH by adding 1 μL sample to pH strip (Hydrion DRJ pH 1‐12, Micro Essential Laboratory cat. no. DJ‐910).
24. Centrifuge at 1000 RCF for 5 minutes. Collect flow through and reload on to StageTip.
25. Transfer the stage tips that contains bounds peptides into a new collection tube for elution of peptides using the abovementioned elution buffers (containing 25 mM ammonium acetate and varying % of ACN). Centrifuge for 1000 RCF for 2‐5 minutes until all liquid has flowed through.
26. Transfer the stage tips to the next collection tube and add the next elution buffer. Repeat this step 7 times until all the peptides have been eluted. Centrifuge at 1000 RCF for approximately 5 minutes until all liquid has flowed through. Aim for ~20 μL/min flow rate. The final flow through is fraction 8.
27. To decrease total instrument time needed for peptide analysis, fractions 1, 7 and 8 were concatenated, leaving six total fractions.
**CRITICAL STEP**: Samples were combined to increase total MS time availability and to ensure each fraction had approximately the same amount of material. Concatenation strategies will be sample dependent.
**PAUSE POINT**: Samples can be stored at −80°C for 1 year.
28. Quantify fractions using the BCA assay (ThermoFisher Scientific) and calculate volume needed for 1 μg for sample injection.

### 7.3 Data acquisition and analysis (timing: 90 minutes per fraction, 9 hours per sample plus additional time for data analysis)

1. Resuspend fractionated peptide samples in 0.1% (vol/vol) formic acid and place into deactivated glass vials designed for proteomics analysis (TruView LCMS Certified Clear Glass, Waters SKU: 186005668CV).
2. Inject up to 1 μg of peptides per sample using a nanoAcquity system (Waters, Milford, MA) and separate peptides on the analytical column maintained at 35°C. Desalt all peptide samples using a trapping column (ACQUITY UPLC M‐Class Symmetry C18 Trap Column, 100 Å, 5 μm, 180 μm × 20 mm) for 5 minutes using Buffer A (99% H_2_O, 1% acetonitrile), and separate peptides using an analytical column (ACQUITY UPLC Peptide BEH C18 column, 130 Å, 1.7 μm, 75 μm × 250 mm) using Buffer B (5.0% to 32.5% acetonitrile gradient over 74 minutes), followed by Buffer B 60% acetonitrile over 6 minutes, at a flow rate of 300 nL/min.
3. Proteomics data acquisition files (Raw files) are analyzed using the Andromeda search engine in MaxQuant with the Mouse Uniprot Database. For all database searches, set missed cleavages to 3, set cysteine carbamidomethylation as a fixed modification and oxidation of methionine residues, N‐terminal acetylation (protein) and deamidation (NQ) were as variable modifications, with a maximum number of modifications per peptide set to 5 and peptide length specified as ≥6. Precursor mass deviation set to 20 ppm and 4.5 ppm for the first and main searches, respectively. Fragment mass deviation set to 20 ppm. For filtering, assign protein and peptide level false discover rate to 0.01 (1%, for each) and decoy databases to revert. Finally, enable the match between runs algorithm and leave all remaining parameters as default.
4. Bioinformatics analyses can be performed using multiple software packages and we recommend using the packages that accompanied MaxQuant called Perseus. Load in protein lists, generated from MaxQuant as text files, in Perseus. Remove proteins that were identified only by site, reverse sequences and contaminants using the filter function. To further filter the proteomics dataset, remove quantified proteins that were only found in one replicate using the valid value filter in Perseus. Categorically annotate each replicate sample a unique name to identify each sample type using the categorical row annotator. Perform a two‐sample *t*‐test to determine if the mean of each group is significantly different, generating a list of proteins, their fold changes and *P*‐values for further downstream analysis.
5. Export list of proteins from Perseus using the export matrix function or by copy and pasting the matrix in a text or Excel document for further downstream analysis.

## 8 METABOLOMICS

### 8.1 Metabolite extraction (timing: ~45 minutes)

Overview of metabolomics is provided in Figure 4B.

1. For metabolite extraction, transfer tissue to Navy RINO screw‐cap tubes (Next Advance, cat. no. NAVYR1) and add 300 μL cold acetonitrile to each sample tube.
2. Homogenize using the Bullet Blender Storm (Next Advance, cat. no. BBY24M) set to Time: 5, and Power: 12, two times.
*Note:* We found that using the Bullet Blender was the most efficient method for multiple samples. However, this step is for simple tissue homogenization which could be done using a mortar and pestle with liquid nitrogen to freeze the tissue or a tissue homogenizer.
3. Remove the metal beads from RINO tubes using a strong magnet slid upwards slowly along the outside of the tube.
4. Immediately place tubes into −20°C freezer for 20 minutes to allow precipitation of proteins.
5. Cool microcentrifuge to 4°C while samples are in freezer.
6. Centrifuge samples at 4°C for 5 minutes at 14 000 RCF.
7. Remove supernatant and transfer samples to new 1.5 mL microcentrifuge tubes.

**PAUSE POINT**: Samples can be stored at −80°C for up to 6 months.

### 8.2 Sample preparation (timing: ~ 5 hours)

Total volume of solvent required for protein precipitation will depend on number of samples to be analyzed. Acetonitrile (ACN) is the organic solvent used in this protocol for protein precipitation. One hundred and fifty microliters of acetonitrile solvent containing internal standards will be added to 50 μL of each sample for protein precipitation. We recommend making 1.2 times the required amount of precipitation solvent.

1. To prepare the precipitation solvent, add chlorpropamide, atenolol‐d7, flurazepam, and DL‐2‐aminoheptanedioic acid to the appropriate volume of LC‐MS grade acetonitrile to achieve concentrations of 1.1 μg/mL, 500 ng/mL, 50 ng/mL, and 17.5 μg/mL, respectively. This solution should be prepared fresh.
*Note:* Chlorpropamide, atenolol‐d7, flurazepam, and DL‐2‐aminoheptanedioic acid will serve as the internal standards in the experiment. In our experience, using these internal standards allows analysis of positive and negative ionization for reverse phase chromatography.
2. Thaw samples on ice. Identical sample preparation was done for both AF and plasma samples.
3. Aliquot 50 μL from each sample into 1.5 mL microcentrifuge tubes.
4. Add 150 μL of ice‐cold acetonitrile solvent containing internal standards into each plasma aliquot tube for protein precipitation (3:1 acetonitrile: plasma vol/vol).
5. Vortex tubes for 30 seconds, then incubate at −20°C for 20 minutes.
6. Centrifuge at 14 000 RCF for 10 minutes.
7. Remove and transfer 150 μL supernatant from samples into new 1.5 mL microcentrifuge tubes, being careful not to disturb the pellet/residue at the bottom.
8. Prepare a new set of 1.5 mL microcentrifuge tubes (one for each sample) and add 80 μL of LC‐MS grade water to each.
9. Transfer 20 μL of supernatant into corresponding tubes containing water (1 in 5 (vol/vol) dilution in water).
10. Vortex the dilution for 10 seconds.
11. Pool a small volume from each water dilution tube (see note below) into a separate 1.5 mL microcentrifuge tube. This pooled sample will be used for quality control and will be injected at regular intervals throughout the metabolomics run.
**CRITICAL STEP**: The amount taken from each diluted sample will depend on the number of samples. As large metabolomics runs take a long time and the pooled sample will be injected frequently, you must ensure there is adequate volume to last the entire analytical run. We recommend pooling enough from each sample to reach a total volume of 300 μL or more.
12. Transfer the remaining contents of the water dilution tubes and the pooled sample into vials designed for MS (Deactivated Clear Glass 12 × 32 mm Screw Neck Total Recovery Vials, Waters SKU: 186000384DV) and cap the vials.
13. Ensure there are no air bubbles in the total recovery vials. If any air bubbles are present, gently tap the bottom of vials on the benchtop to remove the bubbles.
14. Place sample vials and pooled sample vial in 48‐well plates and place the plates into the UPLC sample manager for injection.

### 8.3 LC‐MS/MS acquisition (timing: 11 minutes per sample plus controls)

1. Set injection volume of 2 μL for plasma metabolomics, 5 μL for AF tissue metabolomics.
2. Randomize the sample injection order for both plasma and disc runs but keep it identical between ionization modes (ie, negative ionization mode for plasma is the same order as positive ionization mode).
3. Inject pooled sample 5 times at the very beginning of the run, and then after every five sample injections.

**CRITICAL STEP**: Assign group numbers (G1, G2, G3, etc) for each of your experimental groups, taking note of how you have assigned them. When making your sample list for the analysis, include the date of analysis and assigned group numbers in the file names for each sample (ie, Aug23_2019_6MonthKO1_Disc_G3_Pos).

### 8.4 Data analysis

1. Copy the project folder from run, which contains the “Data” folder (Waters *.raw* data files), to the computer where analysis will be completed.
*Note:* We use a separate computer to analyze the data so other samples can be analyzed by LC‐MS while data analysis is occurring. Data analysis can be performed on the same computer as acquisition if desired.
2. Open the script “Convert Waters MSe file to mzData file” (Supporting Information File 1) in RStudio. This script uses the *convert.waters.raw* package (which will need to be installed) to convert *.raw* data files to *.mzData* files. Set the input folder to the full file path name of the “Data” folder and set the output folder to the same file path name of the “Data” folder, but with “/converted” at the end. This will create a subfolder within “Data” called “converted” and put all the converted files into it.
**CRITICAL STEP**: If using Windows and copying the file path name into RStudio, make sure the slashes separating the directories are forward slashes (“/”) and not backslashes (“\”). R uses forward slashes to denote separate directories, whereas Windows uses backslashes.
3. Run the script.
4. Once the script is finished, create a subfolder in “converted” called “Neg.” Move all .*mzData* files from the negative ionization mode into the “Neg” subfolder. Within the “Neg” subfolder, create subfolders for each experimental group, including the pooled run (ie, G1, G2, G3, Pool). Place all data files into their appropriate subfolders according to sample group.
**CRITICAL STEP**: Ensure that each file has been put into the correct group folder. The script will not run as intended if this is not the case.

**IPO script**:

The IPO script (Supporting Information File 2) is used to optimize parameters for XCMS data processing for peak picking.

1. In your project folder create another subfolder called “IPO.” Inside “IPO,” create two folders: “IPO RPLC Neg” and “IPO RPLC Pos.”
2. Copy the *.mzData* data files of all pooled injections from negative ionization mode, minus the first five pooled injections, into the “IPO RPLC Neg” folder created in the step above. Repeat for positive ionization mode pooled files into “IPO RPLC Pos.”
3. Change the “setwd” and “save. image” parameters in the IPO script to the appropriate target folders.
4. Run IPO script.
5. Copy the parameters generated by IPO into a word document.

**XCMS script**:

XCMS is designed to provide automated processing of LC‐MS metabolomics data.

1. In your project folder, create another subfolder called “XCMS.” Inside “XCMS,” create two folders: “XCMS RPLC Neg” and “XCMS RPLC Pos.”
2. Copy the contents of (project)/Data/converted/Neg (ie, G1, G2, G3, and Pool folders) to “XCMS RPLC Neg” and the contents of (project)/Data/converted/Pos to “XCMS RPLC Pos.”
3. Open XCMS script in R (Supporting Information File 3), load the XCMS package, and set working directory to the “XCMS RPLC Neg” folder.
4. Paste over the existing “xset” parameters in the script with the new parameters from IPO.
5. Change xset names to xsetP, xsetR, xsetG, xsetF ensuring that each xset command draws from the previous (ie, xsetR draws from xsetP, xsetG draws form xsetR, etc).
6. Run XCMS lines between markers labeled “here.”
7. Load the CAMERA package.
8. Use the next lines of the XCMS script to make an annotated diffreport (annotateDiffreport( ) function, part of the CAMERA package).
9. Continue with the script to generated box plots and extracted ion chromatograms for all detected metabolites with the diffreport( ) function. Before you do this, you will need to denote the column numbers of the annotated diffreport that contain your sample groups. The groups will always start at column 13 (ie, G1, G2, G3, and Pool are columns 13‐16).
10. Repeat steps 3‐9 for the positive ionization (Supporting Information File 4) mode files.
11. Create combined positive‐ and negative‐ion diffreport using the combine XCMS script (Supporting Information File 5). The first part of this script loads in files that were saved in the previous scripts for negative and positive ionization modes (saved just before the CAMERA package was loaded).
12. Follow the rest of the script, and you will generate the files “camAnotNeg.csv” and “camAnotPos.csv”.
13. Use the last script (for combined annotated diffreport) to prepare a final output file for EZInfo (Supporting Information File 6). This script will take your annotated diffreport for negative (camAnotNeg.csv) and positive ionization (camAnotPos.csv), normalize each mode to an internal standard, combine both modes, evaluate the quality control (pooled samples) variability, and reorganize/tidy up the data for upload into EZInfo. The steps for all of these processes are outlined in detail in the script itself.

*Note:* This step can be done outside of R if desired.

### 8.5 EZInfo analysis

1. Proceed to EZInfo analysis to identify potential metabolites‐of‐interest by uploading the EZInfo final sheet from the XCMS scripts to a new EZInfo sheet.
2. Click “View” and change the second row (Pos or Neg) to secondary variable by clicking on drop down arrow, then click “done” at the bottom of the window.
3. Change scaling to “Pareto scaling” by clicking on the “View and change the selected template” hyperlink and changing the “Scale type for x‐variables” to “Pareto”. Ensure that file location and name are correct at the bottom of the window, then click “finish.”
4. Click refresh and scroll down to find principal component analysis (PCA) plot.
5. On the right side of the window, select “color by” and change to “sample group.”
6. Ensure all “pooled” samples are in the center of the plot, if so, delete pooled samples from EZInfo final sheet and repeat above EZInfo analysis steps.
7. Generate Orthogonal Projections to Latent Structures Discriminant Analysis (OPLS‐DA) plot by clicking the box of the groups in the legend that you want to compare. Check off OPLS‐DA box at the bottom of the window and click “done.”
8. Create S‐plot from OPLS‐DA data by clicking “S‐plot” at the top of the window. Change x‐axis to p(loadings).
9. Open a new Excel spreadsheet to copy over important columns for identification of potential metabolites‐of‐interest.
10. Within EZInfo, click on “Tools” tab, click “List” to retrieve p(corr) values, click on the top corner to select all and copy to Excel.
11. Within the S‐plot window in EZInfo select VIP (Variable Importance in Projection), then click “Tools,” then “List” to retrieve VIP values and copy to Excel.
12. Sort the p(corr) and VIP lists by primary ID so that they are in the same order, then cut the VIP column and past next to p(corr). Delete remaining two columns from VIP list to leave 5 columns: Primary ID, PosOrNeg, p(1)P, p(corr), VIP.
13. Filter spreadsheet for p(corr) values greater than 0.4 or lesser than −0.4.
14. Then sort by VIP value. VIP values >0.8 are worth attempting to identify.

### 8.6 Identification of metabolites

1. For each metabolite‐of‐interest use the human metabolome database (HMDB) at hmdb.ca. Specifically, within the “search” tab select “LC‐MS Search.” This window will allow you to enter the *m/z* of each metabolite‐of‐interest. Select the correct ion mode from the drop down menu and select “Unknown” for the Adduct Type. Use a molecular weight tolerance of 0.01 to generate a stringent list of potential metabolite IDs.
2. From the HMDB list, choose potential metabolite IDs by having a low “Delta” score (meaning the actual *m/z* is close to theoretical *m/z*; 0 means exactly the same), a potentially relevant biological function and an adduct that is possible in your sample preparation. (ie, most likely adducts are multiples of hydrogen ions M + H, M − H or M + 2H, and so forth adducts adding H_2_O, or ACN makes sense, sodiated ions, that is, M + Na are also common, potassium or methyl group adducts are extremely unlikely given the ionization energy in our MS method).
3. To better predict the correct metabolite ID from multiple potential options, download the .MOL file from each respective metabolite page on HMDB and compare the fragmentation patterns using Mass Fragment in MassLynx as follows.
4. For each metabolite‐of‐interest, click on it in the S‐plot in EZInfo and select “Variable Trend Plot” to generate a trace at the bottom of the window. Note the sample that has the highest value, which will be used to investigate the peak from the chromatogram.
5. Open sample list in the correct mode (Pos or Neg) in MassLynx and select the sample with the highest value for the metabolite‐of‐interest. Click on “Display” then select “Mass” and type in the accurate mass of the metabolite of interest (from the Excel spreadsheet) into the and second function in the pop‐up window and hit Enter.
6. Click “Tools” and select “Mass Fragment”, in the pop‐up window, select the .MOL file saved from HMDB, then click “OK.”
7. A new tab will open, select the correct ionization mode and click “Submit.”
8. View the possible fragments between the different candidate IDs using this strategy. The lower the number beside a fragment, the more likely it is your metabolite‐of‐interest.
9. Once you have determined some metabolites‐of‐interest, you will need to order analytical standards to validate the metabolite ID using the steps below.

### 8.7 Metabolite validation

To confirm the identity of metabolites of interest, analytical standards must be purchased and run in tandem with experimental samples. If the purchased standard has the same retention time, *m/z*, and fragmentation spectrum as the unidentified target analyte in the experimental samples, it is considered a “level 1” (highest level possible) identification according to a previously defined categorization system of metabolite identification.^91^

**FIGURE 5:**
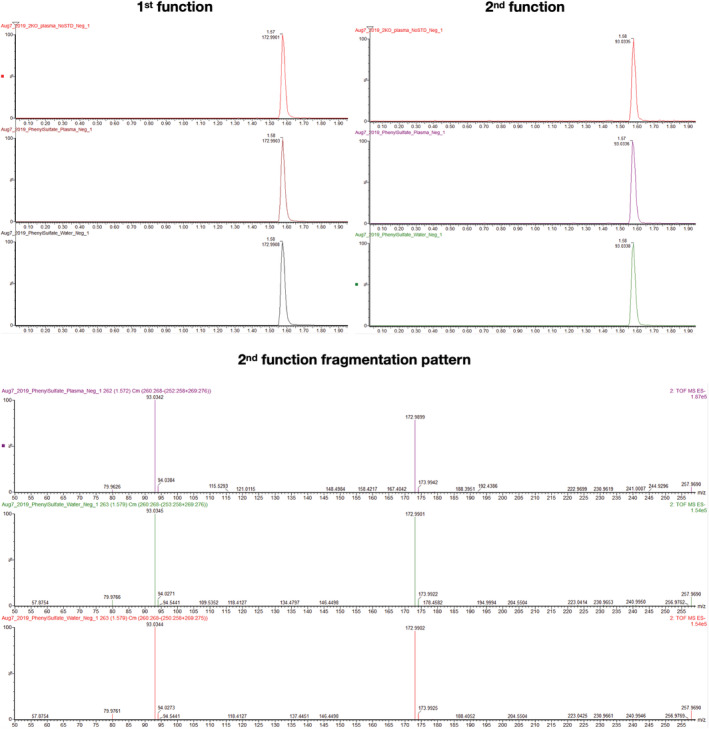
Example validation run for metabolomics using phenyl sulfate identified as a level 1 metabolite

Steps of metabolite validation for one metabolite of interest are as follows:

1. Create a stock solution of your analytical standard. The concentration of stock solution and the solvent used will depend on the quantity of standard purchased and chemical properties of the compound, respectively.
2. For your metabolite of interest, determine which experimental group yielded the highest average signal intensity from the metabolomics run.
3. Pool plasma and/or AF samples from the experimental group with highest average signal intensity. The amount taken from each sample will depend on the number of samples you have in that experimental group. We recommend a total pooled volume of 100 μL.
4. Add your stock standard solution to LC‐MS grade water and plasma/disc pooled samples to achieve a concentration of 100 μM and a total volume of 50 μL, creating a “spiked” water sample, and a “spiked” plasma/disc pooled sample. Prepare a separate aliquot of 50 μL of plasma/disc pooled samples with no standard added (“non‐spiked”).
**CRITICAL STEP**: Use a minimal amount of stock standard solution to avoid drastically altering the composition of biological matrices. We recommend no more than 1% stock (v/v).
*Note:* 100 μM is a recommended starting point that typically results in a strong signal for most metabolites.
5. Continue with sample preparation for “spiked” water, “spiked” plasma/disc pooled sample, and “non‐spiked” plasma/disc pooled sample. Sample preparation from this point forward is identical to steps 3‐13 in the metabolomics sample preparation section, **excluding** step 10.
6. Analyze prepared samples with the same UPLC and MS parameters as described previously.
7. Compare retention time, *m/z*, and fragmentation spectrum between the various “spiked” samples and the “non‐spiked” sample. If all three categories are match, you have level 1 metabolite identification (Figure 5).

## CONFLICT OF INTEREST

The authors state that there are no conflicts of interest for this study.

## AUTHOR CONTRIBUTIONS

Study conception and design: Matthew A. Veras, Yong J. Lim, Miljan Kuljanin, Gilles A. Lajoie, Bradley L. Urquhart, Cheryle A. Séguin.

Acquisition of data: Matthew A. Veras, Yong J. Lim, Miljan Kuljanin.

Analysis and interpretation of data: Matthew A. Veras, Yong J. Lim, Miljan Kuljanin, Gilles A. Lajoie, Bradley L. Urquhart, Cheryle A. Séguin.

## Supporting information


**Data S1** Script files.
