# Supplementary material for: Protocol for parallel proteomic and metabolomic analysis of mouse intervertebral disc tissues
Source: JOR Spine. 2020 Jun 10;3(3):e1099. doi: 10.1002/jsp2.1099 (PMC7524214; doi:10.1002/jsp2.1099)
Supplement: Supplementary file 1 — Data S1 Script files. [file JSP2-3-e1099-s001.zip › JSP2_1099_Supplementary File 6 - Generate Combined Annotated Diffreport R Script.rtf]

##Code for annotated diffreport once it is made######Step 1######Add Pos mz to Neg annotated diffreport and Neg mz to Pos annotated diffreportsetwd("C:/Users/James Lim/Documents/School and Work/Mouse Plasma RPLC Metabolomics/Day 1- 4 Metabolomics Aug 2019")Neg<-read.csv("camAnotNeg.csv") ##Read Neg annotated diffreport with Pos annotationsPos<-read.csv("camAnotPos.csv") ##Read Pos annotated diffreport with Neg annotationsNegIonBoth<-Neg[grep("*Found*", Neg$pos..Mode),] ##subset by ions found in both Neg/PosPosIonBoth<-Pos[grep("*Found*", Pos$neg..Mode),] ##subset by ions found in both Neg/PosNegPosCombine<-data.frame(NegIonBoth$mz, PosIonBoth$mz) ##combine mz found in bothrownames(NegPosCombine)<-NegPosCombine$NegIonBoth.mz ##set row names to first columnrownames(Neg)<-Neg[,2] ##set row names to mz column so same as row names of "NegPosCombine"NegCombine<-merge(Neg, NegPosCombine, by="row.names", all.x = TRUE) ##merge Neg with NegPosCombine keeping all Neg rowsNegCombine[is.na(NegCombine)] <- "" ##remove NAs from NegCombineNegCombine$Row.names<-NULL ##delete Row.names columnNegCombine<-NegCombine[order(NegCombine$X),] ##put Neg back in original orderrownames(NegCombine)<-NegCombine[,1]; NegCombine<-NegCombine[,-1] ##put row names back in Negwrite.csv(NegCombine,"camAnotNeg_addPos.csv") ##export Neg annotated diffreport with Pos mz addedwrite.csv(NegCombine,"NegCombine.csv")rownames(NegPosCombine)<-NegPosCombine$PosIonBoth.mz  ##repeat for Pos moderownames(Pos)<-Pos[,2]PosCombine<-merge(Pos, NegPosCombine, by="row.names", all.x = TRUE)PosCombine[is.na(PosCombine)] <- ""PosCombine$Row.names<-NULLPosCombine<-PosCombine[order(PosCombine$X),]rownames(PosCombine)<-PosCombine[,1]; PosCombine<-PosCombine[,-1]write.csv(PosCombine,"camAnotPos_addNeg.csv")##Stop HERE and proceed to Step 2 and 3 for Neg then do 2 and 3 for Pos########################################################################################################################### NEGATIVE MODE#########################Step 2: Normalize to Internal Standard (ISTD)######Start with Neg Mode####Create "Neg" folder and set it as working directoydir.create("Neg")setwd("Neg")##For NegCombine, Concatenate the mz column and rt column into first column called "mzrt" to read as mz_rt and divide rt by 60 to convert to minutesmzrt<-paste(NegCombine$mz,NegCombine$rt/60,sep = "_")NegCombine<-data.frame(mzrt,NegCombine)##Create a subset object of integrated peaks and "mzrt" column using date as a column name pattern#NegCombineRaw<-data.frame(NegCombine[,"mzrt"],NegCombine[,grep("*Aug23*",colnames(NegCombine))]); names(NegCombineRaw)[1]<-"mzrt"##Check column names of all pooled samples are in ordercolnames(NegCombineRaw[,grep("*Pooled_Plasma_Neg", colnames(NegCombineRaw))])##Determine Column Numbers of first 5 pool samples and removepoolNumbers<-grep("*Pooled_Plasma_Neg", colnames(NegCombineRaw))NegCombineRaw<-NegCombineRaw[,-poolNumbers[14:16]] #For removing Pool 3-5NegCombineRaw<-NegCombineRaw[,-poolNumbers[12]] #For removing Pool 2NegCombineRaw<-NegCombineRaw[,-poolNumbers[1]] #For removing Pool 1##Check column names of to make sure the first 5 pool are gonecolnames(NegCombineRaw)##Create a subset of annotations before peaks and annotations after peaks#NegCombineAnnot<-NegCombine[,-grep("*Aug23*",colnames(NegCombine))] ##subset without integrated peaksNegCombineAnnot1<-NegCombineAnnot[,1:(ncol(NegCombineAnnot)-6)]##subset before peaksNegCombineAnnot2<-NegCombineAnnot[,(ncol(NegCombineAnnot)-5):ncol(NegCombineAnnot)]##subset after peaks##Make the first column the row names then delete first columnrownames(NegCombineRaw)<-NegCombineRaw[,1]; NegCombineRaw<-NegCombineRaw[,-1]##Find ISTD row based on m/z and retention time of ISTD##rownames(NegCombineRaw)##and rename to "ISTD"##ISTDrow<-grep("275.025418227833_2.50791666666667", rownames(NegCombineRaw))rownames(NegCombineRaw)[ISTDrow]<-"ISTD"##Check rownames to confirmrownames(NegCombineRaw)##Note if the number of metabolites is too long, just open the "NegCombineRaw" object## as a new tab to look for and copy the ISTD number, go back to check replacement with "ISTD"##convert to data matrix##df1 = as.matrix(NegCombineRaw)##Divide all rows by ISTD Row (Must find ISTD Row Number)##ISTDMean<-mean(df1["ISTD",], dims = 1) ##calculate mean of all internal standard areasdf1["ISTD",]<-sapply(df1["ISTD",],function(x) x/ISTDMean) ##divide each ISTD by mean ISTDISTD<-t(t(df1)/df1["ISTD",])##Set base file name for Neg Mode DataNegFileName<-"Mouse_Plasma_RPLC_camAnotNeg_addPos_"##export ISTD normalized data for recordswrite.csv(ISTD,paste0(NegFileName,"ISTD.csv"))####Step 3:Evaluate QC Variability####QC<-t(ISTD) ##tranpose ISTD dataframeQCtb <- QC[grep("*Pooled*",rownames(QC)),] ##subset only Pool injectionsQCtb<-t(QCtb) ##transpose againQCsd<-data.frame(apply(QCtb, 1, sd)); names(QCsd)[1] <- "SD"  ##calculate SD for each ionQCmean<-data.frame(rowMeans(QCtb, dims = 1)) ##calculate mean for each ionQCrsd<-QCsd/QCmean; names(QCrsd)[1] <- "RSD" ##calculate RSDQCFinal<-cbind(QCtb,QCrsd) ##add RSD to QCtb dataframewrite.csv(QCFinal,paste0(NegFileName,"QC.csv")) ##export QCFinal for records##combine annotations with ISTD normalized data for rsd filtering####Add RSD values at end of each row##rsd<-data.frame(NegCombineAnnot1,ISTD,NegCombineAnnot2, QCrsd, row.names = 1)##keep peaks with RSD<0.3 or 30%##threshold <- 0.3 ##set threshold##Trim data by the RSD column which is the last column##ISTDTrimmed<-subset(rsd, rsd[,ncol(rsd)] < threshold) ISTDTrimmed = ISTDTrimmed[, -ncol(ISTDTrimmed)] ##remove RSD columnISTDTrimmed[is.na(ISTDTrimmed)] <- "" ##remove NAs##Determine max peak intensity in each row##maxint<-apply(ISTDTrimmed[, grep("*Aug23*",colnames(ISTDTrimmed))], 1, max)ISTDTrimmed<-data.frame(rownames(ISTDTrimmed),ISTDTrimmed,maxint); colnames(ISTDTrimmed)[1]<-"mzrt"##Sort by pcgroup then maxint##ISTDTrimmed<-ISTDTrimmed[order(ISTDTrimmed$pcgroup,ISTDTrimmed$maxint),]##Determine maxint for each group using aggregateISTDTrimmedMAX<-aggregate(ISTDTrimmed$maxint, list(pcgroup = ISTDTrimmed$pcgroup), max); colnames(ISTDTrimmedMAX)[2]<-"maxint"##merge the Max intensity with ISTDTrimmed by pcgroup (peak clusters)##ISTDTrimmedMERGE<-merge(ISTDTrimmed,ISTDTrimmedMAX,by = "pcgroup", all.x = TRUE)##order by pcgroup then maxintISTDTrimmedMERGE<-ISTDTrimmedMERGE[order(ISTDTrimmedMERGE$pcgroup,ISTDTrimmedMERGE$maxint.x),]##movepcgroup to end of dataframe##ISTDTrimmedMERGE<-data.frame(ISTDTrimmedMERGE[,-1],ISTDTrimmedMERGE[,1]); colnames(ISTDTrimmedMERGE)[ncol(ISTDTrimmedMERGE)]<-"pcgroup"write.csv(ISTDTrimmedMERGE,paste0(NegFileName,"ISTD_QCTrimmed.csv")) ##export for recordsNegData<-ISTDTrimmedMERGE ##save as NegData object for later#######Positive MODE#############NOTE SOME OBJECTS ARE REUSED AND THEREFORE OVERWRITTEN FROM NEG ANALYSIS###########Step 2: Normalize to Internal Standard####setwd("..")##Create "Pos" folder and set it as working directoydir.create("Pos")setwd("Pos")##For PosCombine, Concatenate the mz column and rt column into first column called "mzrt" to read as mz_rt and divide rt by 60 to convert to minutesmzrt<-paste(PosCombine$mz,PosCombine$rt/60,sep = "_")PosCombine<-data.frame(mzrt,PosCombine)##Create a subset object of integrated peaks and "mzrt" column using date as a column name pattern#PosCombineRaw<-data.frame(PosCombine[,"mzrt"],PosCombine[,grep("*Aug23*",colnames(PosCombine))]); names(PosCombineRaw)[1]<-"mzrt"##Check column names of all pooled samples are in ordercolnames(PosCombineRaw[,grep("*Pooled_Plasma_Pos*", colnames(PosCombineRaw))])##Determine Column Numbers of first 5 pool samples and removepoolNumbers<-grep("*Pooled_Plasma_Pos", colnames(PosCombineRaw))PosCombineRaw<-PosCombineRaw[,-poolNumbers[14:16]] #For removing Pool 3-5PosCombineRaw<-PosCombineRaw[,-poolNumbers[12]] #For removing Pool 2PosCombineRaw<-PosCombineRaw[,-poolNumbers[1]] #For removing Pool 1##Check column names of to make sure the first 5 pool are gonecolnames(PosCombineRaw)##Create a subset of annotations before peaks and annotations after peaks#PosCombineAnnot<-PosCombine[,-grep("*Aug23*",colnames(PosCombine))] ##subset without integrated peaksPosCombineAnnot1<-PosCombineAnnot[,1:(ncol(PosCombineAnnot)-6)]##subset before peaksPosCombineAnnot2<-PosCombineAnnot[,(ncol(PosCombineAnnot)-5):ncol(PosCombineAnnot)]##subset after peaks##Make the first column the row names then delete first columnrownames(PosCombineRaw)<-PosCombineRaw[,1]; PosCombineRaw<-PosCombineRaw[,-1]##Find ISTD row##rownames(PosCombineRaw)##and rename to "ISTD"##ISTDrow<-grep("274.21438724246_1.19658333333333", rownames(PosCombineRaw))rownames(PosCombineRaw)[ISTDrow]<-"ISTD"##Check rownames to confirmrownames(PosCombineRaw)##convert to data matrix##df1 = as.matrix(PosCombineRaw)##Divide all rows by ISTD Row (Must find ISTD Row Number)##ISTDMean<-mean(df1["ISTD",], dims = 1)df1["ISTD",]<-sapply(df1["ISTD",],function(x) x/ISTDMean)ISTD<-t(t(df1)/df1["ISTD",])##Set base file name for Pos Mode DataPosFileName<-"Mouse_Plasma_RPLC_camAnotPos_addNeg_"##export ISTD normalized data for recordswrite.csv(ISTD,paste0(PosFileName,"ISTD.csv"))####Step 3:Evaluate QC Variability####QC<-t(ISTD) ##tranpose ISTD dataframeQCtb <- QC[grep("*Pooled*",rownames(QC)),] ##subset only Pool injectionsQCtb<-t(QCtb) ##transpose againQCsd<-data.frame(apply(QCtb, 1, sd)); names(QCsd)[1] <- "SD"  ##calculate SD for each ionQCmean<-data.frame(rowMeans(QCtb, dims = 1)) ##calculate mean for each ionQCrsd<-QCsd/QCmean; names(QCrsd)[1] <- "RSD" ##calculate RSDQCFinal<-cbind(QCtb,QCrsd) ##add RSD to QCtb dataframewrite.csv(QCFinal,paste0(PosFileName,"QC.csv")) ##export QCFinal for records##combine annotations with ISTD normalized data for rsd filtering####Add RSD values at end of each row##rsd<-data.frame(PosCombineAnnot1,ISTD,PosCombineAnnot2, QCrsd, row.names = 1)##keep peaks with RSD<0.3 or 30%##threshold <- 0.3 ##set threshold##Trim data by the RSD column which is the last column##ISTDTrimmed<-subset(rsd, rsd[,ncol(rsd)] < threshold) ISTDTrimmed = ISTDTrimmed[, -ncol(ISTDTrimmed)] ##remove RSD columnISTDTrimmed[is.na(ISTDTrimmed)] <- "" ##remove NAs##Determine max peak intensity in each row##maxint<-apply(ISTDTrimmed[, grep("*Aug23*",colnames(ISTDTrimmed))], 1, max)ISTDTrimmed<-data.frame(rownames(ISTDTrimmed),ISTDTrimmed,maxint); colnames(ISTDTrimmed)[1]<-"mzrt"##Sort by pcgroup then maxint##ISTDTrimmed<-ISTDTrimmed[order(ISTDTrimmed$pcgroup,ISTDTrimmed$maxint),]##Determine maxint for each group using aggregateISTDTrimmedMAX<-aggregate(ISTDTrimmed$maxint, list(pcgroup = ISTDTrimmed$pcgroup), max); colnames(ISTDTrimmedMAX)[2]<-"maxint"##merge the Max intensity with ISTDTrimmed by pcgroup##ISTDTrimmedMERGE<-merge(ISTDTrimmed,ISTDTrimmedMAX,by = "pcgroup", all.x = TRUE)##order by pcgroup then maxintISTDTrimmedMERGE<-ISTDTrimmedMERGE[order(ISTDTrimmedMERGE$pcgroup,ISTDTrimmedMERGE$maxint.x),]##movepcgroup to end of dataframe##ISTDTrimmedMERGE<-data.frame(ISTDTrimmedMERGE[,-1],ISTDTrimmedMERGE[,1]); colnames(ISTDTrimmedMERGE)[ncol(ISTDTrimmedMERGE)]<-"pcgroup"write.csv(ISTDTrimmedMERGE,paste0(PosFileName,"ISTD_QCTrimmed.csv")) ##export for recordsPosData<-ISTDTrimmedMERGE ##save as PosData object for later####Step 4: Combine Mode and Filter Adducts#######If you want to 1. Filter for Adducts and/or 2. Filter metabolites that ionize in pos and neg, keeping the more sensitive mode##setwd("..") ##setwd up one folder##make dataframe of NegIonBoth, maxint.y and pcgroup for NegDataNegToMergePos<-data.frame(NegData$NegIonBoth.mz,NegData$maxint.y, NegData$pcgroup); names(NegToMergePos)[1]<-"IonInBoth"NegToMergePos[NegToMergePos==""]<-NA ##set blank spaces to NANegToMergePos<-subset(NegToMergePos,!is.na(IonInBoth)) ##remove NA in "IonInBoth"Column##make dataframe of NegIonBoth (used to merge with NegData above), PosIonBoth, maxint.y and pcgroup for PosDataPosToMergeNeg<-data.frame(PosData$NegIonBoth.mz, PosData$PosIonBoth.mz,PosData$maxint.y, PosData$pcgroup); names(PosToMergeNeg)[1]<-"IonInBoth"PosToMergeNeg[PosToMergeNeg==""]<-NAPosToMergeNeg<-subset(PosToMergeNeg,!is.na(IonInBoth))NegPosMerge<-merge(NegToMergePos,PosToMergeNeg, by = "IonInBoth", all = TRUE) ##merge Neg and Pos together by IonInBoth##Determine Neg ions that are lower than Pos ions to be removedtheMaxNegtoRemove<-data.frame(subset(NegPosMerge, NegPosMerge$NegData.maxint.y<NegPosMerge$PosData.maxint.y))names(theMaxNegtoRemove)[1]<-"NegIonBoth.mz"##Combine NegData and Neg Ions to remove then export to neg folder for records##NegRemoveRaw<-merge(NegData,theMaxNegtoRemove, by="NegIonBoth.mz", all.x = TRUE) ##merge Neg and the ions to remove sheetsNegRemoveRaw[is.na(NegRemoveRaw)] <- "" ##replace NAs with spaceNegRemoveRaw<-as.data.frame(append(NegRemoveRaw, NegRemoveRaw[1], after = ncol(NegRemoveRaw)-4)) ##move column merged (column 1) to end of data sheetNegRemoveRaw<-NegRemoveRaw[,-1] ## delete first columnwrite.csv(NegRemoveRaw,"Neg/NegRemoveRaw.csv", row.names = FALSE)##You can use this exported spreasheet to check if the correct ions were removed for the pos/neg merge because it contains all of themNegFinal<-NegData[!NegData$pcgroup %in% theMaxNegtoRemove$NegData.pcgroup,] ##remove pcgroups in MaxNegtoRemove from Neg##Determine Pos ions that are lower than Neg ions to be removedtheMaxPostoRemove<-data.frame(subset(NegPosMerge, NegPosMerge$NegData.maxint.y>NegPosMerge$PosData.maxint.y))names(theMaxPostoRemove)[3]<-"PosIonBoth.mz"##Combine PosDataFiltered and Pos Ions to remove then export to Pos folder for records##PosRemoveRaw<-merge(PosData,theMaxPostoRemove, by="PosIonBoth.mz", all.x = TRUE)PosRemoveRaw[is.na(PosRemoveRaw)] <- "" ##replace NAs with spacePosRemoveRaw<-as.data.frame(append(PosRemoveRaw, PosRemoveRaw[1], after = ncol(PosRemoveRaw)-1))PosRemoveRaw<-PosRemoveRaw[,-1]write.csv(PosRemoveRaw,"Pos/PosRemoveRaw.csv", row.names = FALSE)PosFinal<-PosData[!PosData$pcgroup %in% theMaxPostoRemove$PosData.pcgroup,]PosFinal[is.na(PosFinal)] <- ""##Export Pos/Neg Trimmed data for recordswrite.csv(NegFinal, paste0("Neg/",NegFileName,"PosTrim.csv"), row.names = FALSE)write.csv(PosFinal, paste0("Pos/",PosFileName,"NegTrim.csv"),row.names = FALSE)##Filter Trimmed Data for Adducts (Emily Note: A LOT of metabolites are lost here)NegFinalFiltered<-subset(NegFinal, NegFinal$maxint.x == NegFinal$maxint.y)PosFinalFiltered<-subset(PosFinal, PosFinal$maxint.x == PosFinal$maxint.y)##Export Pos/Neg ADDUCT Trimmed data for recordswrite.csv(NegFinalFiltered, paste0("Neg/",NegFileName,"PosTrim_AdductFiltered.csv"), row.names = FALSE)write.csv(PosFinalFiltered, paste0("Pos/",PosFileName,"NegTrim_AdductFiltered.csv"),row.names = FALSE)####Step 5: Create EZInfo sheet for Pos and Neg Combined#####NegEZInfo<-t(data.frame(NegFinalFiltered$mzrt, NegFinalFiltered[,grep("*Aug23",colnames(NegFinalFiltered))]))colnames(NegEZInfo)<-NegEZInfo[1,] ##make first row the column namesNegEZInfo<-NegEZInfo[-1,]##delete first rowPosORNeg<-rep("Neg",ncol(NegEZInfo))NegEZInfo<-rbind(PosORNeg,NegEZInfo)NegEZInfo<-data.frame(rownames(NegEZInfo),NegEZInfo); rownames(NegEZInfo)<-NULL; colnames(NegEZInfo)[1]<-"SampleName"PosEZInfo<-t(data.frame(PosFinalFiltered$mzrt, PosFinalFiltered[,grep("*Aug23*",colnames(PosFinalFiltered))]))colnames(PosEZInfo)<-PosEZInfo[1,] ##make first row the column namesPosEZInfo<-PosEZInfo[-1,]##delete first rowPosORNeg<-rep("Pos",ncol(PosEZInfo))PosEZInfo<-rbind(PosORNeg,PosEZInfo)PosEZInfo<-data.frame(rownames(PosEZInfo),PosEZInfo); rownames(PosEZInfo)<-NULL; colnames(PosEZInfo)[1]<-"SampleName"##Check that samples are in same order for Pos and Neg Modedata.frame(NegEZInfo$SampleName,PosEZInfo$SampleName)##Delete Pos mode SampleName columna and combine Neg and Pose dataframesPosEZInfo$SampleName<-NULLNegPosCombineEZInfo<-data.frame(NegEZInfo,PosEZInfo)##String split to obtain group labelsEZInfoGroups<-strsplit(as.character(NegPosCombineEZInfo$SampleName),"_")##string splitEZInfoGroups[1]<-NULL##remove PosORNegEZInfoGroups<-as.factor(sapply(EZInfoGroups, "[[",3)) ##obtain group labels, note that the column number for group labels will depending on your file naming systemEZInfoGroups<-gsub("Neg","Pooled",EZInfoGroups) ##replace Neg with PoolEZInfoGroups<-append("",EZInfoGroups)##insert blank cell in list for PosOrNeg##Add SampleGroup labels##NegPosCombineEZInfo<-data.frame(append(NegPosCombineEZInfo, list(SampleGroups=EZInfoGroups), after = 1)); rownames(NegPosCombineEZInfo)<-NULL##Remove date and Mode from SampleName ColumnNegPosCombineEZInfo$SampleName<-gsub("Aug23_2019_","",NegPosCombineEZInfo$SampleName)NegPosCombineEZInfo$SampleName<-gsub("_Neg","",NegPosCombineEZInfo$SampleName)##Export EZinfo csvwrite.csv(NegPosCombineEZInfo, "Mouse_Plasma_RPLC_CombinedMode_EZinfo_NoAdductFilter_Final.csv", row.names = FALSE)save.image(file="RPLC Combined Annotated Diffreport R Environment.RData")
